# Effects of ankle and knee braces on leg stiffness during hopping

**DOI:** 10.1186/1757-1146-7-S1-A108

**Published:** 2014-04-08

**Authors:** Hiroaki Hobara, Yoshiyuki Kobayashi, Tomoya Ueda, Masaaki Mochimaru

**Affiliations:** 1 National Institute of Advanced Industrial Science and Technology, Tokyo, 135-0064, Japan; 2 Tokyo University of Science, Chiba, 278-8510, Japan

## 

**Figure 1:**
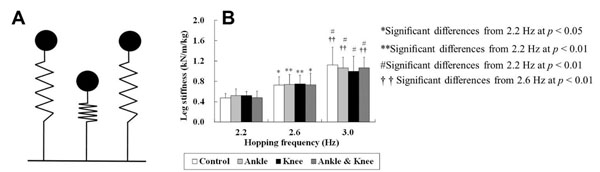
A: Spring-mass model for hopping. This model consists of a body mass and a massless linear spring supporting the body mass. The model is shown at the beginning of the ground contact phase (left), the middle of ground contact phase (middle), and at the end of ground contact phase (right). B: Comparison of *K*leg among brace conditions in three hopping frequencies.

In a spring-mass model (Figure 1-A), the stiffness of the leg spring (leg stiffness; *K*leg) is thought to be an important factor in musculoskeletal performance in hopping, running and jumping [1]. Despite the fact that many athletic activities are performed with joint stabilizers, little is known about the *K*leg with ankle and/or knee braces. A previous study demonstrated that neither ankle taping nor bracing affected the *K*leg during hopping at 3.0 Hz [2]. However, it remains unclear if this constant *K*leg exists or changes at other hopping frequencies. The purpose of this study was to more extensively investigate the effect of ankle and knee braces on the *K*leg over a range of hopping frequencies.

Ten male participants performed one-legged hopping in place, matching metronome beats at 2.2, 2.6, and 3.0 Hz. Based on a spring-mass model, we calculated *K*leg using an inertial sensor (Myotest ®, Myotest SA, Switzerland). Commercially-available ankle and knee braces (Ankle Guard-soft and Knee Guard-Ligament3, ALCARE, Japan) were used to constrain these joints, respectively.

Statistical analysis revealed the existence of a significant main effect of hopping frequency (*F*(1.22, 10.97)= 48.16, *p*< 0.01; Figure 1-B) on *K*leg but no significant main effect of brace conditions (*F*(3.00, 27.00)= 0.15, *p*= 0.926), nor a significant interaction between hopping frequency and brace conditions (*F*(6.00, 54.00)= 0.94, *p*= 0.472) on *K*leg. These results indicate that neither ankle nor knee bracing affects the *K*leg in a range of hopping frequency.
